# Changes in adiposity, physical activity, cardiometabolic risk factors, diet, physical capacity and well-being in inactive women and men aged 57-74 years with obesity and cardiovascular risk – A 6-month complex lifestyle intervention with 6-month follow-up

**DOI:** 10.1371/journal.pone.0256631

**Published:** 2021-08-25

**Authors:** Laila A. Hopstock, Trygve S. Deraas, Andre Henriksen, Torsten Martiny-Huenger, Sameline Grimsgaard

**Affiliations:** 1 Faculty of Health Sciences, Department of Community Medicine, UiT The Arctic University of Norway, Tromsø, Norway; 2 Faculty of Health Sciences, Department of Psychology, UiT The Arctic University of Norway, Tromsø, Norway; Pennington Biomedical Research Center, UNITED STATES

## Abstract

A key challenge in lifestyle interventions is long-term maintenance of favorable lifestyle changes. Middle-aged and older adults are important target groups. The purpose of this analysis was to investigate changes in adiposity, physical activity, cardiometabolic risk factors, diet, physical capacity, and well-being, in inactive middle-aged and older women and men with obesity and elevated cardiovascular disease risk, participating in an interdisciplinary single-arm complex lifestyle intervention pilot study. Participants were recruited from the population-based Tromsø Study 2015–2016 with inclusion criteria age 55–74 years, body mass index (BMI) ≥30kg/m^2^, sedentary lifestyle, no prior myocardial infarction and elevated cardiovascular risk. Participants (11 men and 5 women aged 57–74 years) underwent a 6-month intervention of two 1-hour group-sessions per week with instructor-led gradually intensified exercise (endurance and strength), one individual and three 2-hour group counselling sessions with nutritionist (Nordic Nutrition Recommendations) and psychologist (Implementation intention strategies). We investigated changes in adiposity (weight, BMI, body composition, waist circumference), physical activity (self-reported and via physical activity trackers), cardiometabolic risk factors (blood pressure, HbA1c, blood lipids), diet (intake of energy, nutrients, foods), physical capacity (aerobic capacity, muscle strength), and psychological well-being, measured at baseline and end-of-intervention, using mean-comparison paired t-tests. Further, we investigated self-reported healthy lifestyle maintenance six months after end-of-intervention, and monthly changes in daily step count, moderate-to-vigorous physical activity (MVPA) and total energy expenditure. From baseline to end-of-intervention, there was a mean decrease in weight, BMI, fat mass, waist circumference, intake of total- and saturated fat, and increase in lean mass, lateral pulldown and leg press. We detected no changes in mean levels of physical activity, cardiometabolic risk factors or well-being. Six months after end-of-intervention, 25% responded healthy lifestyle achievement and maintenance, while objectively measured physical activity remained unchanged. The results are useful for development of a protocol for a full-scale trial.

**Trial registration:** The study was registered at www.ClinicalTrials.gov registry (NCT03807323).

## Introduction

Obesity, today a worldwide epidemic, is a preventable condition caused by an imbalance between energy consumed, i.e. dietary intake, and energy expended, i.e. physical activity [1,2]. Physical inactivity is one of the leading risk factors for death worldwide, and 1 in 3 adults in high-income countries are insufficiently physically active [3]. Improvements in physical activity levels and dietary habits, leading to weight loss and reduced disease risk, can be achieved by successful lifestyle interventions [2]. However, people frequently return to baseline physical activity levels and weight after end-of-intervention. Thus, a key challenge is long-term maintenance of lifestyle changes [2,4–7], often defined by keeping the attained physical activity level [4] or weight loss [5] after intervention, typically for at least one year [5,7].

Life expectancy is increasing, and the world’s population is ageing [8], thus, middle-aged and older adults are important target groups for long-term maintenance of lifestyle interventions, including improvements in physical activity levels [9]. Lifestyle interventions typically consist of one or several elements such as exercise, dietary restrictions and/or psychological interventions. Combinations of intervention elements lead to increased long-term weight loss [10]. Although lifestyle interventions are shown to be safe and effective in middle-aged and older adults [11–14], few complex lifestyle interventions target this group [11,12].

The purpose of this analysis was to investigate changes in adiposity, physical activity, cardiometabolic risk factors, diet, aerobic capacity, muscle strength, and psychological well-being, in inactive middle-aged and older women and men with obesity and elevated cardiovascular risk, participating in a complex lifestyle intervention pilot and feasibility study. Our long-term aim is to develop and test a complex lifestyle intervention, the RESTART (Re-inventing Strategies for healthy Ageing; Recommendations and Tools) trial, to investigate maintenance of improvements in physical activity and adiposity in middle-aged and older adults.

## Materials and methods

### Design

The study was designed as a multi-factorial, interdisciplinary, mixed-methods, single-arm exploratory study of a complex lifestyle intervention. Assessment of feasibility (i.e. recruitment, data collection, intervention, responsiveness, adherence and adverse events) and participant experiences are described elsewhere [15,16]. Study method details are found in the study protocol (S1 Appendix).

### Sample and setting

**Fig 1:**
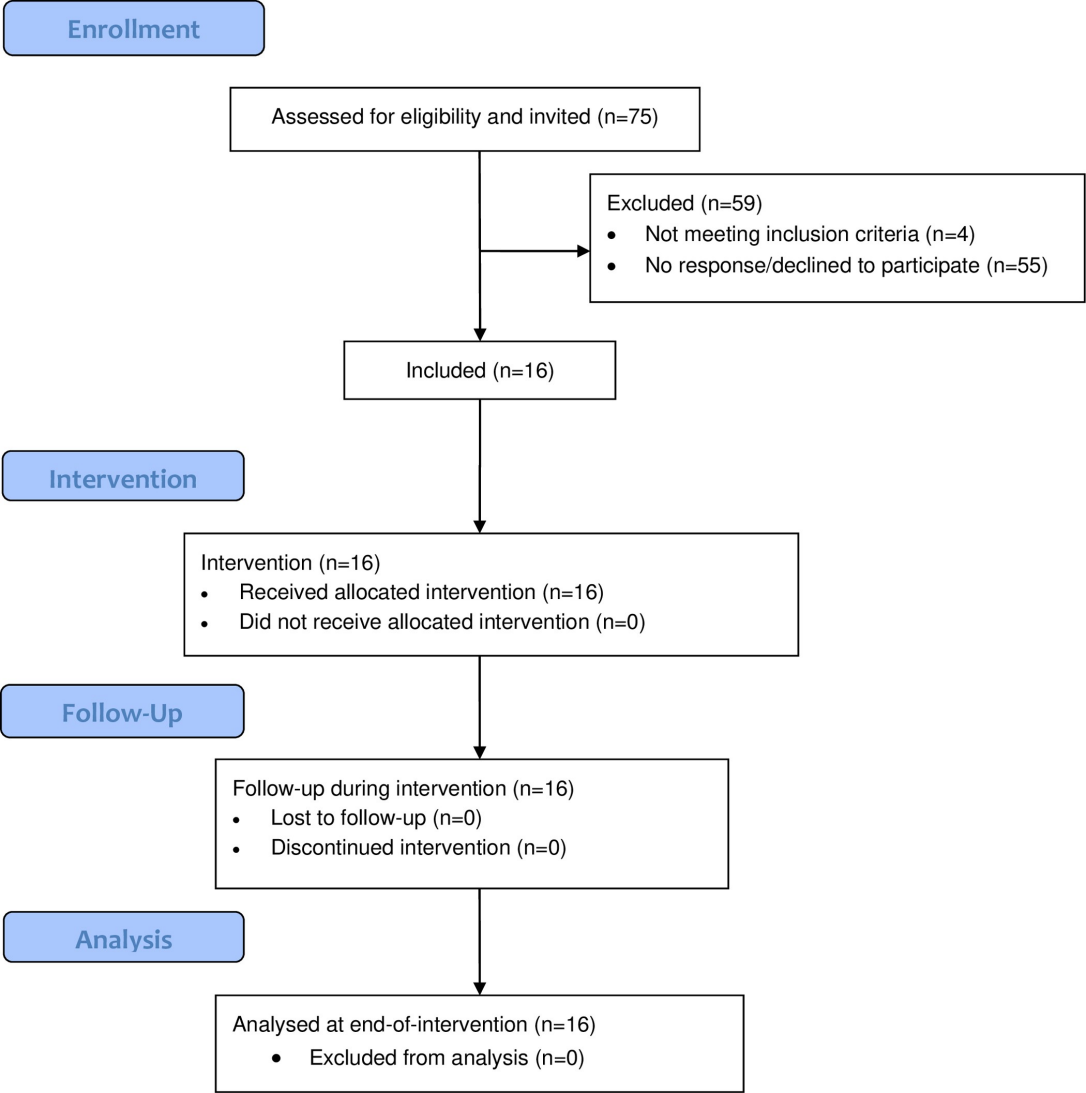
CONSORT flowchart (modified to fit a non-randomized pilot study). The RESTART pilot study 2017–18.

Tromsø, the largest municipality in Northern Norway (population 76,000) is situated above the Arctic Circle at 69°N and has large seasonal variations in weather conditions and daylight with a dark season (November-January) and midnight sun (May-July) period. Study participants were recruited of the population-based Tromsø Study [17], from a randomly selected sample who had previously participated in the seventh survey (Tromsø 7, 2015–2016). In Tromsø 7, all inhabitants aged 40 years or older were invited, of which 21,083 women and men aged 40–99 years participated (attendance 65%) [18]. Inclusion criteria for the current study were; age 55–74 years, body mass index (BMI) ≥30kg/m^2^, self-reported physical activity level inactive as defined by the Saltin and Grimby questionnaire [19], no prior myocardial infarction, and elevated 10-year risk of incident cardiovascular disease risk as defined by NORRISK2 [20]. After invitation of a randomly selected sample from Tromsø 7 (N = 75, 76% men) and initial screening (n = 20 responded, i.e. 27%) including telephone interviews (n = 4 excluded), clinical examinations and physical function tests, a final sample of 11 men and 5 women aged 57–74 years were included (Fig 1). As described elsewhere [16], the final sample did not differ from the non-responders except for lower smoking prevalence.

#### Ethics approval and consent

The study was approved by the Regional Committee of Medical and Health Research Ethics (REC North reference 2017/1100). The participants gave written informed consent.

#### Trial registration

The study was registered at the www.ClinicalTrials.gov registry (NCT03807323).

### Intervention

All participants underwent a 6-month intervention of two 1-hour group sessions per week with instructor-led gradually intensified exercise (focusing on endurance (bicycle spinning) and strength, balance and flexibility (bodyweight/dumbbells exercises, resistance training)), one 1-hour individual and three 2-hour group counselling sessions with nutritionist (focussing on general and practical food knowledge, shopping and cooking based on the Nordic Nutrition Recommendations (NNR) [21]) and three 2-hour group counselling sessions with a psychologist (implementing and teaching a behavioural self-regulation strategy; Implementation Intentions [22]). Details on the study intervention are described elsewhere [15,16]. Adherence to all components of the intervention was high, of which mean attendance for the twice-a-week exercise sessions was 70% [15]. Study start was 25^th^ of September 2017, intervention period October 2017 –March 2018, with follow-up to 4^th^ of October 2018.

### Outcome measures

Primary outcomes included improvement in adiposity and physical activity level at end-of-intervention, and maintenance of healthy lifestyle and physical activity levels six months after end-of-intervention. Secondary outcomes included improvement in cardiometabolic risk factors, diet, aerobic capacity, muscle strength, and psychological well-being, at end-of-intervention.

### Assessment

All outcome measurements were performed at one week before start-of-intervention (baseline) and one week after intervention end (end-of-intervention) with standard methods, by trained personnel. Furthermore, six months after end-of-intervention (end-of-follow-up), i.e. 12 months after baseline, information on healthy lifestyle achievement and maintenance (open question: *“Did you form new habits*, *and did you keep them*?*”*) were obtained from individual semi-structured in-depth qualitative follow-up interviews aimed to explore participant experiences. Details about the method and results from the qualitative interviews used in this study are described elsewhere [16]. In addition, daily accelerometer-measured physical activity was obtained from a consumer-based physical activity tracker from baseline, throughout the intervention to end-of-follow-up. Details of about the method and results from use of the activity tracker are described elsewhere [16]. Demographic data on marital status (cohabitant yes/no) and educational level (university/university college education yes/no) were collected via questionnaire. Baseline examination was performed in September 2017, end-of-intervention in March 2018, and end-of follow-up September 2018.

#### Adiposity

Adiposity was assessed as body weight, BMI (body weight in kilograms (kg) divided by body height in meters squared (m)^2^), waist circumference measured in centimetres (cm) at the umbilical level with a measuring tape, and body fat and lean mass percentage measured by dual-energy X-ray (Lunar GE Prodigy Advance, GE Medical Systems, USA). Weight satisfaction (*“Are you satisfied with your current weight*?*”*), ideal weight (*“What is your ideal weight*?*”*) was assessed via questionnaire.

#### Physical activity

Self-reported physical activity level (*“Reading*, *TV watching or other sedentary activity”* (inactive), *“Walking*, *cycling*, *or other forms of exercise at least 4 hours per week”* (light), *“Participation in recreational sports*, *heavy gardening*, *etc at least 4 hours per week”* (moderate), *“Participation in intensive exercise or competitive sports regularly several times a week”* (vigorous)) was measured with the Saltin and Grimby leisure-time physical activity questionnaire [19], and sitting by a slight modification (hours/weekday, hours/weekend day) of the International Physical Activity Questionnaire (IPAQ) [23] were collected via questionnaire. In addition, participants were asked to continuously wear a Polar M430 (Polar Electro Oy, Finland) physical activity tracker watch on their non-dominant wrist, from baseline, throughout the intervention period, to six months after end-of-intervention, totalling 12 months of physical activity monitoring. Daily physical activity data including step count steps/day, moderate-to-vigorous physical activity (MVPA) minutes (min)/day and total energy expenditure kilocalories (kcal)/day, were synchronised and collected from smart phones. The activity tracker was used for continuous objective physical activity monitoring and not as an intervention element, therefore the Polar M430 user-feedback messages were disabled. Details of the use of the activity tracker in this study are described elsewhere [16].

#### Cardiometabolic risk factors

Systolic and diastolic blood pressure were measured on the right upper arm with a properly sized cuff three times with one-minute intervals after two minutes rest using an oscillometric digital automatic device (Dinamap ProCare 300 monitor, GE Healthcare, Norway), of which the mean of the two last readings was used in the analysis. Non-fasting blood samples were processed immediately after collection and analysed for HbA1c and total-, low-density lipoprotein (LDL)- and high-density lipoprotein (HDL) cholesterol. All blood sample analyses were performed at the Department of Laboratory Medicine at the University Hospital of North Norway (ISO certification NS-EN ISO 15189). Smoking status (current/former/never smoker) was assessed via questionnaire.

#### Diet

Data on food intake were collected via a previously validated food frequency questionnaire (FFQ) [24]. From the FFQ, total energy intake, percentage of total energy intake (E%) for energy-providing nutrients (fat, saturated fat, protein, carbohydrates, sugar, alcohol, and gram (g) for fibre), food (g/day for selected main food groups including vegetables, fruit and berries, fish and shellfish, cakes, sweets and sugar) and alcohol (g/day) intake were calculated using Kostberegningssystemet (KBS version 7.3, University of Oslo, Norway), which is based on the Norwegian food composition tables from 2014 and 2015 [25].

#### Aerobic capacity and muscle strength

Resting heart rate (beats/min) was measured three times with one-minute intervals after two minutes rest with an oscillometric digital automatic device (Dinamap ProCare 300 monitor, GE Healthcare, Norway). The third measurement was used in the analysis. Physical exercise capacity was assessed as VO_2peak_ (millilitre (ml)/min/kg and litre (L)/min) measured during walking/running on a treadmill (Woodway GmbH, weil am Rhein, Germany) according to the test protocol by Rognmo et al [26] using a chest-worn heart rate monitor (Polar RS400, Polar Oy, Finland) and a face mask connected to a ergo spirometry system mixing chamber system (Cosmed K5, Cosmed SRL, Italy) positioned on the participants´ back. The average of the three highest 10-second measurements was set as VO_2peak_. Maximal muscular strength was tested as the heaviest weight the participant could handle during one repetition maximum (1-RM) in three exercises according to the test protocol by Kraemer et al [27]. The exercise consisted of 1) 1-RM leg press (Impulse IT7006 45° Hack Squat), 2) Seated 1-RM lateral pulldown (Technogym Selection Pro Lat Vertical Traction), and 3) Seated incline 1-RM chest press (Technogym Pure Strength Incline Chest Press).

#### Psychological well-being

Via validated questionnaire tools, we assessed self-efficacy via General Self-Efficacy Scale [28], self-esteem via Rosenberg’s Self-Esteem Scale [29], satisfaction with life via Satisfaction With Life Scale [30], symptoms of anxiety and depression via Hopkins Symptom Checklist-10 [31], and global health on a visual analogue scale similar to EQ-VAS [32].

### Analysis

We assessed changes by presentation of mean change with standard deviations (SD), *p*-values (significance threshold set at 0.05) and 95% confidence intervals (CI) from paired t-tests or Wilcoxon matched-pair signed-rank test and interquartile range (25^th^ and 75^th^ percentile) for continuous variables (normally and non-normally distributed, respectively), and percentages with numbers for categorical variables without statistical tests. Shapiro-Wilk test and visual examination of histograms were performed to assess normality in continuous variables (mean change). To account for multiple testing, the false discovery rate was controlled at 0.10 using the Benjamini–Hochberg procedure. For the self-esteem scale, the initial Cronbach’s alpha was very low (baseline 0.57, end-of-intervention 0.19). Two items were identified as being mostly negatively correlated with the remaining items. These items were removed, and the reported values represent the remaining eight items. In the same scale, one participant had missing information on item 10, which was replaced with the median of the remaining items from the same participant. General tendencies in psychological well-being were explored in a forest plot with CI’s (vertical line representing the null effect), where, to make all well-being scales comparable, the CI’s of the anxiety and depression scale was reversed, and the global health scale was scaled down by a factor of 10. Monthly means with CI of steps/day, MVPA min/day and total energy expenditure kcal/day were explored in graphs, of which baseline measures was from one week only (the week before intervention start). No power calculation was performed prior to data collection, as the original aim of the main study was to test the study feasibility and not the effect of the intervention itself. Due to missing or invalid information at either baseline, end-of-intervention or six months after end-of-intervention, data from one or more participants were excluded prior to analysis. However, to investigate study feasibility [15] we aimed to enroll minimum 12 participants (actual size of an exercise intervention sub-group in a full-scale trial). Thus, also for the current analysis, an a priori minimum number of participants (participants with complete data from both baseline and end-of-intervention, or end-of-intervention to end-of-follow-up, respectively) was set to 12 participants. No criteria for adherence to the intervention elements were used in the analyses. Statistical analyses were performed per protocol using Stata version 16 (StataCorp. 2019. Stata Statistical Software: College Station, TX, StataCorp LLC).

## Results

### Baseline characteristics and data completeness

In the final sample of 11 men and 5 women, median age was 65 years, 60% had tertiary education, 19% were smokers, 20% had hypertension, 50% had hypercholesterolemia and 100% reported to be were unsatisfied with their current body weight (Table 1).

**Table 1:** Participant characteristics at baseline. The RESTART pilot study 2017–18.

Characteristics	Baseline
Age, years	66.1 (5.8)
Male sex, %	68.8 (11)
Cohabitant, %	62.5 (10)
University education, %	62.5 (10)
Current daily smoker, %	18.8 (3)
Hypertension, %	18.8 (3)
Hypercholesterolemia, %	50.0 (8)
Weight satisfaction, %	0.0 (0)

Values are mean (standard deviation) or percentage (number).

Hypertension, blood pressure ≥140/90 mmHg; Hypercholesterolemia, total serum cholesterol ≥ 5.0 mmol/L.

Missing information on smoking: One participant.

Missing information on weight satisfaction: Three participants.

Data at baseline were complete with the following exceptions; self-reported physical activity (n = 2), smoking (n = 1), anxiety/depression (n = 2), self-reported health (n = 2), weight satisfaction and ideal weight (n = 3), VO_2peak_ (n = 1), lateral pulldown (n = 1), self-efficacy (n = 2), self-esteem (n = 2), satisfaction with life (n = 2), anxiety/depression (n = 2) and global health (n = 4). Data at end-of-intervention were complete with the following exceptions; self-reported physical activity (n = 1), weight satisfaction and ideal weight (n = 1), blood pressure (n = 1), diet (n = 1), VO_2peak_ (n = 2), chest press (n = 1), lateral pulldown (n = 1) and leg press (n = 2). Complete data (defined as ≥ 10 hours of wear time/day) from daily physical activity trackers were available for all participants up to end-of-intervention, thereafter, we lacked information from two participants for the further six months of follow-up. Complete data from FFQ was available for 15 participants. All participants completed the end-of-follow up interview. Participants with missing information were excluded from the respective analyses prior to analysis. Due to outlier values (recorded body weight gain of 8.7 kg, >3 times higher than the second highest weight gain from baseline to end-of-intervention), analyses of change in adiposity were performed with and without the outlier.

### Lifestyle changes from baseline to end-of-intervention

#### Adiposity and physical activity

From baseline to end-of-intervention, mean body weight decreased by 2.8 (3.8) kg (95% CI: -4.92, -0.68, p = 0.0135), BMI 1.0 (1.3) kg/m^2^ (95% CI: -1.68, -0.28, p = 0.0092), total body fat mass 2.0 (1.8) kg (95% CI: -2.98, -0.98, p = 0.0008), waist circumference 4.2 (3.4) cm (95% CI: -6.07, -2.33, p = 0.0003) and difference between actual weight and ideal weight by 4 (3.8) kg (p = 0.0007), while mean total body lean mass increased by 1.9 (1.8) kg (95% CI: -7.01, -2.43, p = 0.0010) (Table 2). Mean percentage weight loss was 2.6%. The proportion reporting being sedentary decreased from 72% to 33%, self-reported weekday sitting-time decreased (0.0368) and there was a non-significant increase in activity tracker measured daily number of steps, minutes in MVPA and total energy expenditure (Table 3).

**Table 2:** Change in adiposity from baseline to end-of-intervention. The RESTART pilot study 2017–18.

	Baseline	End of intervention	95% CI	P-value*	P-value**
Body weight, kg	106.2 (14.9)	103.4 (14.2)	-4.92, -0.68	0.0135	0.0962
Body mass index, kg/m^2^	35.7 (5.5)	34.7 (5.0)	-1.68, -0.28	0.0092	0.1144
Body fat mass, %	40.2 (6.5)	38.2 (6.3)	-2.98, -0.98	0.0008	0.0021
Body lean mass, %	56.9 (6.2)	58.8 (5.9)	0.92, 2.92	0.0010	0.0020
Waist circumference, cm	117.7 (11.9)	113.5 (12.0)	-6.07, -2.33	0.0003	0.0008
Weight satisfaction, %	0 (0)	20 (3)	NA	NA	NA
Actual vs ideal weight, kg	25.6 (16.4)	20.9 (15.3)	-7.01, -2.43	0.0007	0.0007

Values are means (standard deviations) or percentages (numbers) and confidence intervals for difference between measurements.

CI, confidence interval; Actual vs ideal weight, difference between measured weight and self-reported ideal weight in kilograms.

*Paired t-test for difference between baseline and end of intervention values. One participant was removed before analysis due to outlier values.

**Paired t-test for differences between baseline and end of intervention values with outlier included.

Missing information on weight satisfaction: Three participants at baseline, one participant at end-of-intervention.

Missing information on ideal weight: Three participants.

**Table 3:** Change in physical activity from baseline to end-of-intervention. The RESTART pilot study 2017–18.

	Baseline	End of intervention	95% CI/p25, p75	P-value*
Steps, count/day	7582 (3591)	7974 (2964)	-1727, 2511	0.6987
MVPA, min/day	124 (84)	139 (60)	-29.28, 58.40	0.4898
TEE, kcal/day	2795 (695)	2856 (573)	-244.25, 345.25	0.6571
MVPA ≥150 min/week, %	43.8 (7)	43.8 (7)	NA	NA
Sedentary, %	72 (10)	33 (5)	NA	NA
Sitting weekday, hours/day	9.7 (3.6)	7.7 (2.9)	-4.5, -1.0	0.0368
Sitting weekend, hours/day	7.8 (3.0)	6.7 (2.1)	-2.6, 0.5	0.1618

Values are means (standard deviations) or percentage (number) and confidence intervals or 25th and 75th percentiles for difference between measurements.

CI, confidence interval; p25, 25th percentile; p75, 75th percentile; MVPA: Moderate-vigorous physical activity; TEE, Total energy expenditure; kcal, kilocalories.

Steps, MVPA and total energy expenditure measured with Polar M430 physical activity tracker.

Sedentary, Leisure-time physical activity level as defined by the Saltin & Grimby questionnaire.

Sitting, Sitting as defined by a modified version of the International Physical Activity Questionnaire.

*Paired t-test or Wilcoxon matched-pair singed rank test for difference between baseline and end of intervention values.

Missing information on self-reported physical activity level: Two participants at baseline, one participant at end-of-intervention.

#### Cardiometabolic risk factors, diet, physical capacity and psychological well-being

From baseline to end-of-intervention there were non-significant changes in blood pressure, blood lipid levels and HbA1c, and one out of the three baseline smokers quitted smoking (S1 Table). The mean decrease in intake of total fat was 3.7 (5.6) E% (95% CI: -6.77, -0.60, p = 0.0225) and saturated fat 1.7 (2.1) E% (95%CI: -2.81, -0.52, p = 0.0074) (S2 Table). For intakes of other nutrients (S2 Table) and foods (S3 Table), there were non-significant changes. The mean increase in VO_2peak_ was 2.1 (3.3) ml/kg/min (95% CI: 0.14, 4.07, p = 0.0381), 1-RM lateral pulldown 5.4 (4.0) kg (95% CI: 3.03, 7.68, p = 0.003) and 1-RM leg press 77 (40.7) kg (95% CI: 53.70, 100.62, p<0.0001) (S4 Table). Following Benjamini–Hochberg adjustment, the change in V0_2peak_ related to body weight was not longer statistically significant. There were no changes in resting heart rate or chest press. There were non-significant changes in self-esteem, self-efficacy, satisfaction with life, prevalence of symptoms of anxiety or depression, or global health (S5 Table). Descriptively, all scales indicated better well-being at end-of-intervention compared to baseline (S1 Fig).

### Maintenance of healthy lifestyle

**Fig 2:**
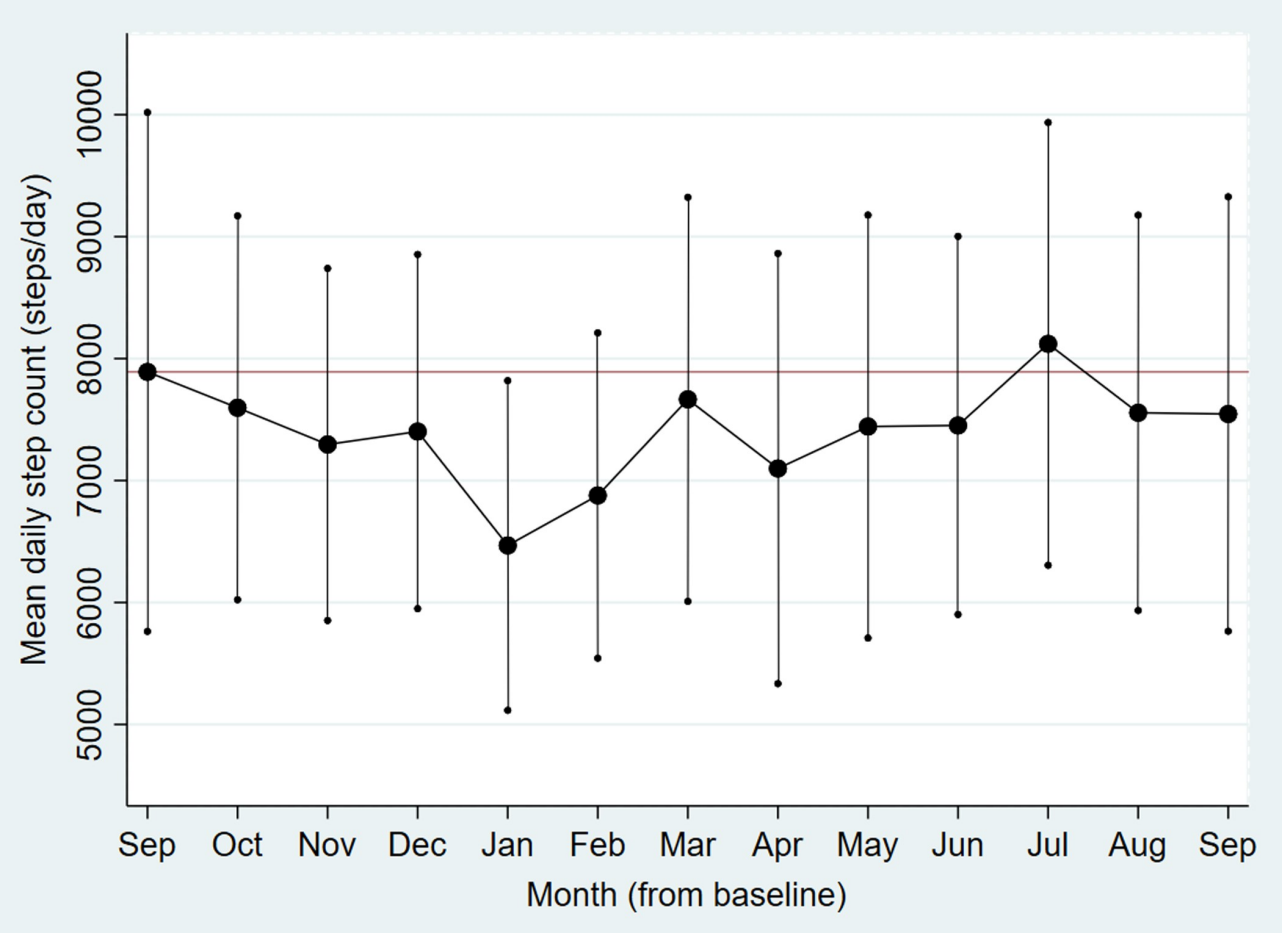
Monthly mean daily step count from baseline to end-of-follow-up. The RESTART pilot study 2017–18.

**Fig 3:**
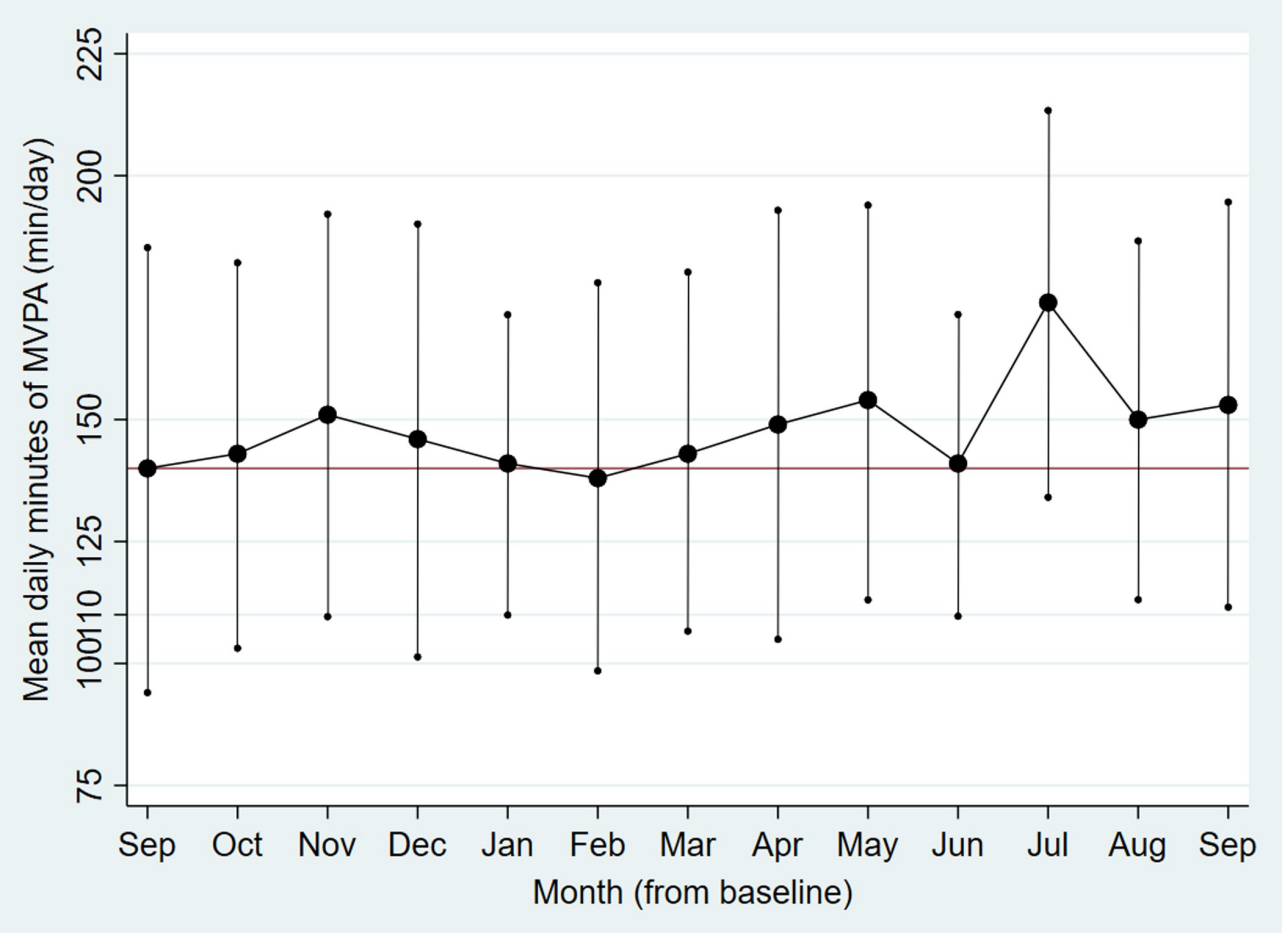
Monthly mean daily minutes in moderate-to-vigorous physical activity from baseline to end-of-follow-up. The RESTART pilot study 2017–18.

**Fig 4:**
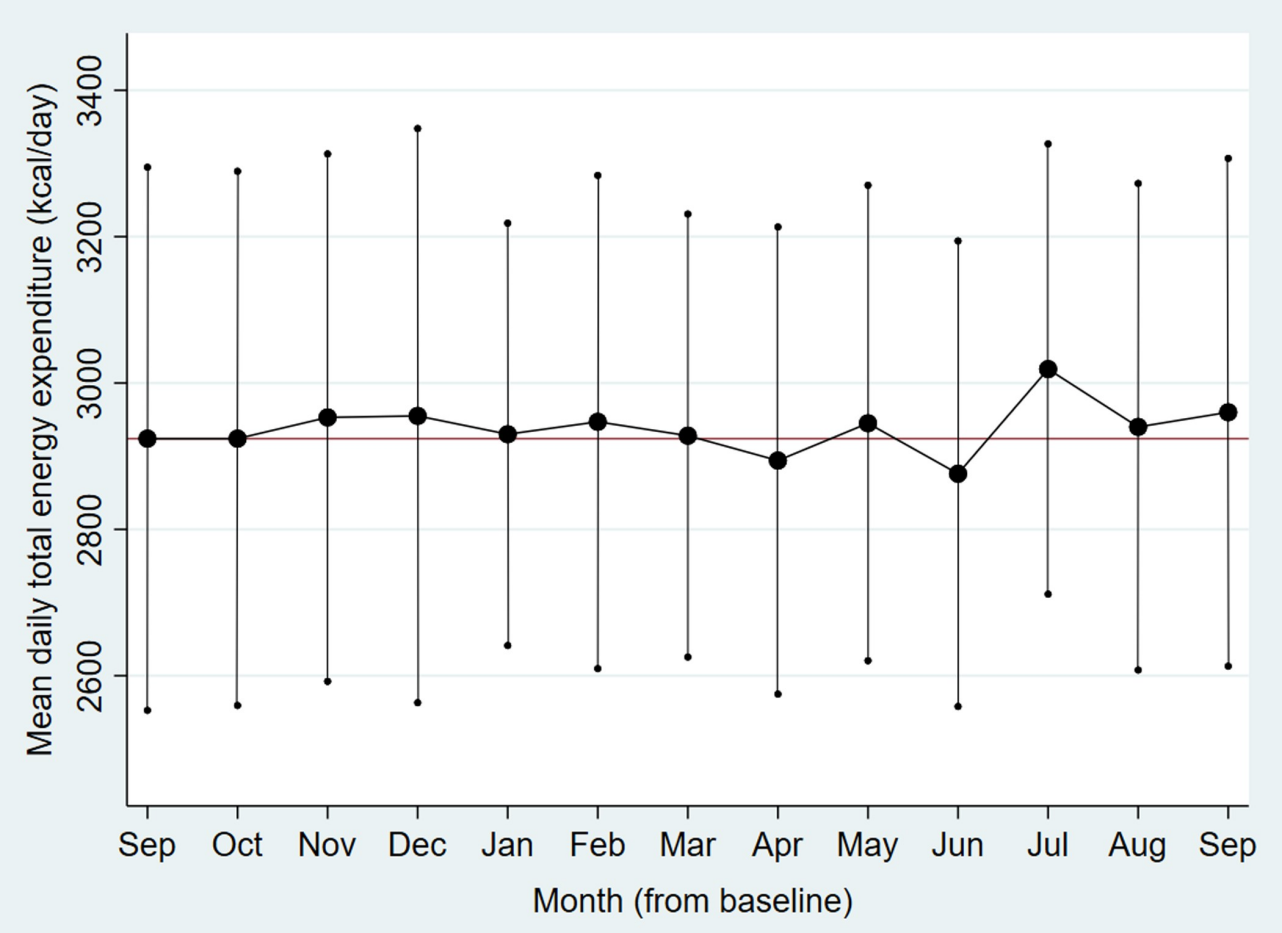
Monthly mean daily total energy expenditure from baseline to end-of-follow-up. The RESTART pilot study 2017–18.

Descriptively, monthly means of activity tracker measured daily number of steps, minutes in MVPA and total energy expenditure fluctuated from baseline (September) to end-of-intervention (March) and further up to end-of-follow-up (September) (Figs 2–4). Lower mean step count was found in the dark season (November-January) and during seasonal flu months (January-February), and peaked in the main summer holiday month (July). Mean minutes of MVPA and total energy expenditure followed a somewhat different pattern with initial increase during the first part of the intervention, but with the same decline in January/February and peak in July. From end-of-intervention to end-of-follow-up there was a non-significant increase in activity tracker measured daily minutes in MVPA and total energy expenditure and decrease in number of steps (Table 4). At the end-of-follow-up interview, 25% of the participants responded that they had achieved favourable lifestyle changes after participating in the study, and that they had maintained these lifestyle modifications.

**Table 4:** Change in physical activity from end-of-intervention to end-of-follow-up, and prevalence of healthy lifestyle achievement and maintenance at end-of-follow-up. The RESTART pilot study 2017–18.

	End of intervention	End of follow-up	95% CI/p25, p75	P-value*
Steps, count/day	7666 (2869)	7546 (3085)	-874, 583	0.5936
MVPA, min/day	143 (64)	153 (72)	-15.8, 35.1	0.4275
TEE, kcal/day	2928 (524)	2960 (601)	-108.0, 105.0	0.6602
MVPA ≥150 min/week, %	50 (7)	50 (7)	NA	NA
Healthy lifestyle, %	NA	25 (4)	NA	NA

Values are means (standard deviations), or percentages (number) and confidence intervals or 25th and 75th percentiles for difference between measurements.

CI, confidence interval; p25, 25th percentile; p75, 75th percentile; MVPA, Moderate-vigorous physical activity, TEE, Total energy expenditure; kcal: Kilocalories.

Healthy lifestyle, Self-reported achievement and maintenance of healthy lifestyle from individual semi-structured qualitative follow-up interview.

Steps, MVPA and total energy expenditure measured with Polar M430 physical activity tracker.

*Paired t-test or Wilcoxon matched-pair singed rank test for difference between end of intervention and end of follow up values.

Missing information from physical activity tracker: Two participants.

Dots are monthly mean number of steps per day, vertical lines are corresponding confidence intervals. The horizontal line represents the baseline mean.

Dots are monthly mean minutes (min) of moderate-to-vigorous physical activity (MVPA) per day, vertical lines are corresponding confidence intervals. The horizontal line represents the baseline mean.

Dots are monthly mean total energy expenditure in kilocalories (kcal) per day vertical lines are corresponding confidence intervals. The horizontal line represents the baseline mean.

## Discussion

From baseline to end-of-intervention, we found favourable changes in the primary outcome adiposity (all measures) and in the secondary outcomes physical capacity (lateral pulldown and leg press) and diet (total and saturated fat intake), but non-significant minor changes in the primary outcome physical activity. At end-of-follow-up, objectively measured physical activity remained unchanged compared to end-of-intervention, and one in four participants responded having achieved and maintained a new healthy lifestyle.

Meta-analyses and systematic review studies examining the effect of various lifestyle interventions in middle-aged and older participants have found successful weight loss [10–12], and increase in objectively measured physical activity [9] or reduction in sedentary behaviour [13]. Less consistent effects are observed for cardiometabolic risk factors [12], physical capacity [12], diet [10], and psychological well-being [12], which is in line with our findings.

The lack of increase in objectively measured physical activity in the present study contrasts findings in other studies including a review study of older adults [9], and may have several explanations. Firstly, we cannot rule out that participants may have increased their physical activity at baseline screening due to measurement awareness and/or social desirability. Furthermore, the observed decrease in numbers of steps, and increase in MVPA minutes per day throughout the intervention and to end-of-follow-up may reflect a shift from light physical activity (mainly walking) to MVPA (including bicycle spinning during intervention). Wrist worn accelerometers do not capture well physical activity on a bicycle [33], especially when using stationary bikes [34]. Another factor is seasonal weather variation including icy or snowy streets that limit outdoor walking from October to April in Tromsø, as well as the seasonal flu in January-February, both which in part may explain the observed decrease in steps in these periods. Seasonal variation in physical activity including lower prevalence of walking and higher total energy expenditure in winter have previously been described in high-precision accelerometer data from a cross-sectional study in a large free-living population [34]. For a full-scale trial, adaptations may be needed to suit local needs and preferences [35]. The lack of increase in objectively measured physical activity contrasts the decrease in adiposity and increase in endurance, strength and self-reported physical activity with high adherence to the exercise sessions. This inconsistency may reflect that we did not capture a possible shift in physical activity pattern over time in this sample. A recent systematic review and meta-analysis of randomized controlled exercise trials in older adults [14] found no studies using objectively measured physical activity to investigate long-term changes in physical activity levels (defined as six months or more). Thus, we cannot compare these findings with other studies.

The observed decrease in adiposity and fat intake accompanied with increase in lean mass (i.e. muscle mass) and weight satisfaction along with improvements in aerobic capacity and muscle strength indicate that participants achieved favourable lifestyle changes, although within the lower end of clinical significance. Further, a subsample reported that they had maintained life style changes at 12 months. A meta-analysis have shown that combinations of intervention elements lead to larger intervention effects [10]. Thus, the dietary changes could be an effect of the minimal nutrition intervention, but could also be a health behaviour effect of participating in a complex intervention, as with the finding of one of the three smokers quitting smoking. Validated standardised methods were used to measure effects. Nevertheless, we cannot rule out that the observed change in aerobic capacity and muscle strength may partly be explained by test familiarisation in a sample of inactive participants.

Psychological well-being measures as self-efficacy, self-esteem, satisfaction with life, symptoms of anxiety and depression, or global health remained largely unchanged. This finding indicate that the intervention did not have negative psychological effects, although regular exercise was challenging and potentially stressful as the participants were confronted with health- or body-related deficits during the intervention. To the contrary, albeit not statistically significant, all measures indicate a change into the direction of higher well-being. A review study of weight-loss interventions and psychological well-being including measures of self-esteem, symptoms of depression, body image and health-related quality of life among adults up to the age of 65 years [36] showed consistent improvements in psychological outcomes with and sometimes also without weight-loss. However, few weight-loss interventions studies investigated psychological well-being as a primary outcome, and the relationship between weight-loss and psychological outcomes needs further investigation [36].

There is a need for studies investigating long-term effects of lifestyle interventions in middle-aged and older adults. A recent review study [37] found that the effects in scaled-up obesity interventions were typically 75% or less of the effects reported in pre–scale-up efficacy trials. Meta-analysis and review studies of lifestyle intervention effects in older adults report overall low methodological quality and scarcity in studies of complex interventions [12,13]. It is therefore important to conduct high-quality pilot- and feasibility studies among older adults prior to full-scale trials. In this pilot and feasibility study, our study sample consisting of middle-aged and older adults underwent a minimal-to-moderate dietary- and psychology intervention, and a resource-demanding exercise intervention. Lessons learned from analyzing study feasibility [15] and effect of study regimen (the current analysis), are that a full-scale randomised controlled trial is safe and feasible, but needs refinement and improvement of intervention elements and measurement methods.

### Strengths and limitations

This analysis has several limitations. The aim of the main study was to pilot the study regimen and evaluate study feasibility and not the effect of the intervention elements as such. Thus, the small sample size, cases with missing data, and lack of a priori power calculations for the studied outcomes, are major limitations for this analysis. Also, the low inclusion proportion may be associated with selection of highly motivated participants, which is likely to influence the results. Therefore, although our results in general are in line with previous findings, we are careful drawing conclusions based on our findings alone. A potential limitation is the use of data from a consumer-based activity tracker without openly available information about how participant’s outcomes are estimated. However, this is a limitation of most consumer based activity trackers. Another limitation is the lack of follow-up data on objective measures of adiposity, to study maintenance of weight loss. However, in-depth data from qualitative interviews on achievement and maintenance of a healthy lifestyle one year after baseline were available for all participants. An extended follow-up period beyond the 6 months after intervention would have been ideal, as long-term follow-as is typically defined as one year [5,12]. Finally, test familiarisation cannot be ruled out, although we used validated and standardised methods to measure intervention effects.

A strength of the study was the high participant adherence. Hence, it was possible to study multiple outcomes after a complex intervention including several components, performed in a previously scarcely studied sub-group, using standard validated methods. Another strength was the possibility to follow-up participants with detailed daily information from an activity tracker on several components of physical activity levels. The activity tracker showed high adherence and low-to-moderate participant burden, as described elsewhere [16]. The use of follow-up physical activity data collected by consumer-based activity trackers can be limited by lack of validation against research-based accelerometers, and here we have questioned whether a shift in physical activity patterns possibly can be left undetected. However, in a previously published validation study comparing the PolarM430 (used in the current study) with ActiGraph wGT3X-BT, we found PolarM430 to provide valid measures of total energy expenditure and, although overestimating steps and MVPA, having acceptable properties for monitoring physical activity [38].

## Conclusions

In this analysis of intervention effects in a pilot study, we observed a decrease in adiposity, and fat intake, and increase in strength, but no change in physical activity, cardiometabolic risk factors, other dietary factors or well-being. Further, six months after end-of-intervention, 25% responded to have achieved and maintained a healthy lifestyle. The observed decrease in adiposity and increase in strength but lack of statistically significant changes in physical activity may indicate a shift in physical activity pattern over time that we were insufficiently able to measure by the long-term use of an activity tracker. There is no evidence that the demanding intervention had negative effects on psychological well-being. Together with the findings from the analysis of the study feasibility, and supported by findings in previous studies, these results are useful for development of a protocol for a full-scale trial.

## Supporting information

S1 Fig Change in psychological well-being from baseline to end-of-intervention. The RESTART pilot study 2017–18.
(DOCX)

S1 Table Change in cardiovascular risk factors from baseline to end-of-intervention. The RESTART pilot study 2017–18.
(DOCX)

S2 Table Change in daily total energy intake and proportion of energy-giving nutrients from baseline to end-of-intervention. The RESTART pilot study 2017–18.
(DOCX)

S3 Table Change in daily food intake from baseline to end-of-intervention. The RESTART pilot study 2017–18.
(DOCX)

S4 Table Change in physical capacity from baseline to end-of-intervention. The RESTART pilot study 2017–18.
(DOCX)

S5 Table Change in psychological well-being from baseline to end-of-intervention. The RESTART pilot study 2017–18.
(DOCX)

S1 Appendix Study protocol. (PDF)

## References

[1] World Health Organisation. Obesity and overweight. 2018. [cited 2020 July 1]. Available from: https://www.who.int/news-room/fact-sheets/detail/obesity-and-overweight.

[2] Tappia PS, Defries D. Prevalence, Consequences, Causes and Management of Obesity. In: Tappia PS, Ramjiawan B, Dhalla NS, editor. Pathophysiology of Obesity-Induced Health Complications. Cham: Springer; 2020. pp. 3–22.

[3] World Health Organisation. Physical activity. 2018. [cited 2020 July 1]. Available from: https://www.who.int/news-room/fact-sheets/detail/physical-activity.

[4] Kahlert D. Maintenance of physical activity: Do we know what we are talking about? Prev Med Rep. 2015;2: 178–80. doi: 10.1016/j.pmedr.2015.02.013 26844069 PMC4721385

[5] Wing RR, Phelan S. Long-term weight loss maintenance. Am J Clin Nutr. 2005;82:222S–5S. doi: 10.1093/ajcn/82.1.222S 16002825

[6] Burgess E, Hassmén P, Pumpa KL. Determinants of adherence to lifestyle intervention in adults with obesity: a systematic review. Clin Obesity. 2017;7:123–35. doi: 10.1111/cob.12183 28296261

[7] Montesi L, El Ghoch M, Brodosi L, Calugi S, Marchesini G, Dalle Grave R. Long-term weight loss maintenance for obesity: a multidisciplinary approach. Diabetes Metab Syndr Obes. 2016;9:37–46. doi: 10.2147/DMSO.S89836 27013897 PMC4777230

[8] Partridge L, Deelen J, Slagboom PE. Facing up to the global challenges of ageing. Nature. 2018;561: 45–56. doi: 10.1038/s41586-018-0457-8 30185958

[9] Chase JAD. Interventions to Increase Physical Activity Among Older Adults: A Meta-Analysis. Gerontologist. 2015;55: 706–18. doi: 10.1093/geront/gnu090 25298530 PMC4542588

[10] Johns DJ, Hartmann-Boyce J, Jebb SA, Aveyard P. Diet or exercise interventions vs combined behavioral weight management programs: Systematic review and meta-analysis of direct comparisons. J Acad Nutr Diet. 2014;114: 1557–68. doi: 10.1016/j.jand.2014.07.005 25257365 PMC4180002

[11] Felix HC, West DS. Effectiveness of Weight Loss Interventions for Obese Older Adults. Am J Health Promot. 2013;27: 191–9. doi: 10.4278/ajhp.110617-LIT-259 23286596 PMC3539224

[12] Witham MD, Avenell A. Interventions to achieve long-term weight loss in obese older people: A systematic review and meta-analysis. Age Ageing. 2010;39: 176–84. doi: 10.1093/ageing/afp251 20083615

[13] Aunger JA, Doody P, Greig CA. Interventions targeting sedentary behavior in non-working older adults: a systematic review. Maturitas. 2018;116: 89–99. doi: 10.1016/j.maturitas.2018.08.002 30244786

[14] Sansano-Nadal O, Giné-Garriga M, Brach JS, Wert DM, Jerez-Roig J, Guerra-Balic M, et al. Exercise-Based Interventions to Enhance Long-Term Sustainability of Physical Activity in Older Adults: A Systematic Review and Meta-Analysis of Randomized Clinical Trials. Int J Environ Res Public Health. 2019;16: 2527. doi: 10.3390/ijerph16142527 31311165 PMC6678490

[15] Deraas TS, Hopstock LA, Henriksen A, Morseth B, Sand AS, Njølstad I, et al. Complex lifestyle intervention among inactive older adults with elevated cardiovascular disease risk and obesity. A mixed-method, single-arm feasibility study for RESTART—a randomized controlled trial. Research Square [Preprint]. 2020. [posted 2020 July 14; cited 2020 July 20]. Available from: https://www.researchsquare.com/article/rs-39292/v1 doi: 10.21203/rs.3.rs-39292/v1 PMC8555104 34706777

[16] Henriksen A, Sand A-S, Deraas T, Grimsgaard S, Hartvigsen G, Hopstock L. Succeeding with prolonged usage of consumer-based activity trackers in clinical studies: A mixed method approach. BMC Public Health 2020;20: 1300. doi: 10.1186/s12889-020-09406-w 32854671 PMC7457262

[17] Jacobsen BK, Eggen AE, Mathiesen EB, Wilsgaard T, Njølstad I. Cohort profile: the Tromsø Study. Int J Epidemiol. 2012;41: 961–967. doi: 10.1093/ije/dyr049 21422063 PMC3429870

[18] The Tromsø Study. The Tromsø Study 2020 July 1 [cited 2020 July 1]. Available from: http://www.tromsoundersokelsen.no.

[19] Grimby G, Börjesson M, Jonsdottir IH, Schnohr P, Thelle DS, Saltin B. The “Saltin-Grimby Physical Activity Level Scale” and its application to health research. Scand J Med Sci Sports. 2015;25 Suppl 4: 119–125. doi: 10.1111/sms.12611 26589125

[20] Selmer R, Igland J, Ariansen I, Tverdal A, Njølstad I, Furu K, et al. NORRISK 2: A Norwegian risk model for acute cerebral stroke and myocardial infarction. Eur J Prevent Cardiol. 2017;24: 773–82. doi: 10.1177/2047487317693949 28206819

[21] Nordic Council of Ministers. Nordic Nutrition Recommendations 2012—Integrating nutrition and physical activity. Nord 2014:002. Available from: http://norden.diva-portal.org/smash/get/diva2:704251/FULLTEXT01.pdf.

[22] Gollwitzer PM. Implementation intentions: Strong effects of simple plans. Am Psychol. 1999;54: 493–503.

[23] Craig CL, Marshall AL, Michael S, Bauman AE, Booth ML, Ainsworth BE, et al. International Physical Activity Questionnaire: 12-Country Reliability and Validity. Med Sci Sports Exerc. 2003;35: 1381–95. doi: 10.1249/01.MSS.0000078924.61453.FB 12900694

[24] Carlsen M, Lillegaard I, Karlsen A, Blomhoff R, Drevon C, Andersen L. Evaluation of energy and dietary intake estimates from a food frequency questionnaire using independent energy expenditure measurement and weighed food records. Nutr J. 2010;9: 37. doi: 10.1186/1475-2891-9-37 20843361 PMC2949781

[25] Norwegian Food Safety Authority. Norwegian Food Composition Database 2015. [cited 2020 July 1]. Available from: https://www.matportalen.no/verktoy/the_norwegian_food_composition_table/the_norwegian_food_composition_table-1.

[26] Rognmo Ø, Hetland E, Helgerud J, Hoff J, Slørdahl SA. High intensity aerobic interval exercise is superior to moderate intensity exercise for increasing aerobic capacity in patients with coronary artery disease. 2004;11: 216–22. doi: 10.1097/01.hjr.0000131677.96762.0c 15179103

[27] Kraemer WJ, Ratamess NA, Fry AC, French DN. Strength Training: Development and Evaluation of Methodology. In: Maud PJ, Foster C, editors. Physiological Assessment of Human Fitness2nd ed. Champaign: Human Kinetics; 2006. pp. 119–50.

[28] Lesanger A, Kraft P, Røysamb E. Perceived self-efficacy in health behavior research: Conceptualisation, measurement and correlates. Psychol Health. 2000;15: 51–69.

[29] Rosenberg M. Society and the adolescent self-image. Princeton, NJ: Princeton University Press; 1965.

[30] Diener E, Emmmons RA, Larsen RJ, Griffin S. The Satisfaction With Life Scale. J Personal Assess. 1985;49: 71–5. doi: 10.1207/s15327752jpa4901_13 16367493

[31] Strand BH, Dalgard OS, Tambs K, Rognerud M. Measuring the mental health status of the Norwegian population: A comparison of the instruments SCL-25, SCL-10, SCL-5 and MHI-5 (SF-36). Nord J Psychiatry. 2003;57: 113–8. doi: 10.1080/08039480310000932 12745773

[32] Herdman M, Gudex C, Lloyd A, Janssen MF, Kind P, Parkin D, et al. Development and preliminary testing of the new five-level version of EQ-5D (EQ-5D-5L). Qual Life Res. 2011;20: 1727–36. doi: 10.1007/s11136-011-9903-x 21479777 PMC3220807

[33] Mannini A, Intille SS, Rosenberger M, Sabatini AM, Haskell W. Activity recognition using a single accelerometer placed at the wrist or ankle. Med Sci Sports Exerc. 2013;45: 2193–203. doi: 10.1249/MSS.0b013e31829736d6 23604069 PMC3795931

[34] Willetts M, Hollowell S, Aslett L, Holmes C, Doherty A. Statistical machine learning of sleep and physical activity phenotypes from sensor data in 96,220 UK Biobank participants. Scientific Reports. 2018;8: 7961. doi: 10.1038/s41598-018-26174-1 29784928 PMC5962537

[35] Durlak JA, DuPre EP. Implementation Matters: A Review of Research on the Influence of Implementation on Program Outcomes and the Factors Affecting Implementation. Am J Community Psychol. 2008;41: 327. doi: 10.1007/s10464-008-9165-0 18322790

[36] Lasikiewicz N, Myrissa K, Hoyland A, Lawton CL. Psychological benefits of weight loss following behavioural and/or dietary weight loss interventions. A systematic research review. Appetite. 2014;72: 123–37. doi: 10.1016/j.appet.2013.09.017 24075862

[37] McCrabb S, Lane C, Hall A, Milat A, Bauman A, Sutherland R, et al. Scaling-up evidence-based obesity interventions: A systematic review assessing intervention adaptations and effectiveness and quantifying the scale-up penalty. Obes Rev. 2019;20: 964–82. doi: 10.1111/obr.12845 30868745

[38] Henriksen A, Grimsgaard S, Horsch A, Hartvigsen G, Hopstock LA. Validity of the Polar M430 Activity Monitor in Free-Living Conditions: Validation Study. JMIR Form Res. 2019;3:e14438. doi: 10.2196/14438 31420958 PMC6716339

