# Supplementary material for: Changes in adiposity, physical activity, cardiometabolic risk factors, diet, physical capacity and well-being in inactive women and men aged 57-74 years with obesity and cardiovascular risk – A 6-month complex lifestyle intervention with 6-month follow-up
Source: PLoS One. 2021 Aug 25;16(8):e0256631. doi: 10.1371/journal.pone.0256631 (PMC8386855; doi:10.1371/journal.pone.0256631)
Supplement: S1 Fig — The RESTART pilot study 2017–18. (DOCX) [file pone.0256631.s001.docx]

**S6 Fig. Change in psychological well-being from baseline to end-of-intervention. The RESTART pilot study 2017-18.**


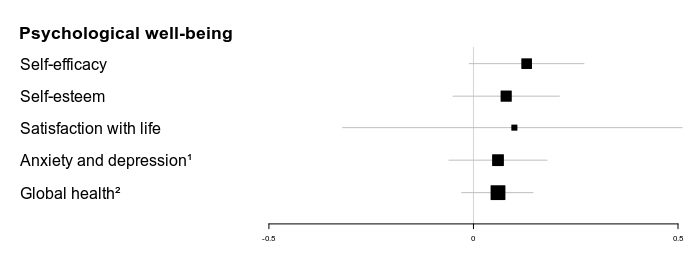


The vertical line represents the null effect.

^1^The confidence interval of the anxiety and depression scale was reversed for comparison.

^2^The global health scale was scaled down by a factor of 10 for comparison.
