# Supplementary material for: Changes in adiposity, physical activity, cardiometabolic risk factors, diet, physical capacity and well-being in inactive women and men aged 57-74 years with obesity and cardiovascular risk – A 6-month complex lifestyle intervention with 6-month follow-up
Source: PLoS One. 2021 Aug 25;16(8):e0256631. doi: 10.1371/journal.pone.0256631 (PMC8386855; doi:10.1371/journal.pone.0256631)
Supplement: S1 Table — The RESTART pilot study 2017–18. (DOCX) [file pone.0256631.s002.docx]

**S1 Table.** **Change in cardiovascular risk factors from baseline to end-of-intervention. The RESTART pilot study 2017-18.**

|  | Baseline | End of intervention | 95% CI/  p25, p75 | P-value* |
| --- | --- | --- | --- | --- |
| Systolic blood pressure, mmHg | 130.7 (13.1) | 128.2 (13.9) | -11.09, 5.97 | 0.5287 |
| Diastolic blood pressure, mmHg | 77.7 (7.1) | 76.9 (8.4) | -5.11, 3.51 | 0.6965 |
| Total cholesterol, mmol/L | 5.17 (0.90) | 5.08 (1.00) | -0.55, 0.35 | 0.4374 |
| LDL cholesterol, mmol/L | 3.46 (1.00) | 3.57 (1.03) | -0.26, 0.48 | 0.5507 |
| HDL cholesterol, mmol/L | 1.22 (0.27) | 1.17 (0.24) | -0.15, 0.05 | 0.3252 |
| Triglycerides, mmol/L | 1.59 (0.55) | 1.7 (0.73) | -0.11, 0.32 | 0.3077 |
| HbA1c, % | 6.0 (0.8) | 5.98 (0.8) | -0.17, 0.08 | 0.4760 |
| Smoking, % | 20.0 (3) | 13.3 (2) | NA | NA |

Values are means (standard deviations) or percentages (numbers) and confidence intervals or 25th and 75th percentiles for difference between measurements.

CI, confidence interval; p25, 25th percentile; p75, 75th percentile; NA, not applicable.

*Paired t-test or Wilcoxon matched-pair singed rank test for difference between baseline and end of intervention values.

Missing information on blood pressure: one participant.

Missing information on smoking: one participant at baseline.
