# Supplementary material for: Changes in adiposity, physical activity, cardiometabolic risk factors, diet, physical capacity and well-being in inactive women and men aged 57-74 years with obesity and cardiovascular risk – A 6-month complex lifestyle intervention with 6-month follow-up
Source: PLoS One. 2021 Aug 25;16(8):e0256631. doi: 10.1371/journal.pone.0256631 (PMC8386855; doi:10.1371/journal.pone.0256631)
Supplement: S2 Table — The RESTART pilot study 2017–18. (DOCX) [file pone.0256631.s003.docx]

**S2 Table.** **Change in daily total energy intake and proportion of energy-giving nutrients from baseline to end-of-intervention. The RESTART pilot study 2017-18.**

|  | Baseline | End of intervention | 95% CI/  p25, p75 | P-value* |
| --- | --- | --- | --- | --- |
| Total energy intake, kJ | 9425 (3681) | 8367 (3175) | -2363, 250 | 0.1048 |
| Fat, E% | 35.4 (5.38) | 31.7 (4.31) | -6.77, -0.60 | 0.0225 |
| Saturated fat, E% | 12.6 (2.72) | 11.0 (2.29) | -2.81, -0.52 | 0.0074 |
| Protein, E% | 17.8 (2.23) | 18.7 (3.59) | -0.60, 2.41 | 0.2173 |
| Carbohydrates, E% | 40.8 (5.82) | 42.9 (4.66) | -1.08, 5.20 | 0.1818 |
| Sugar, E% | 4.8 (3.68) | 3.91 (2.13) | -1.20, 1.10 | 0.6698 |
| Fiber, g/day | 26.4 (10.0) | 28.4 (9.41) | -2.60, 6.48 | 0.3747 |
| Alcohol, E% | 3.7 (3.56) | 3.87 (3.31) | -1.40, 0.40 | 0.6071 |

Values are means (standard deviations) and confidence intervals or 25th and 75th percentiles for difference between measurements.

CI, confidence interval; p25, 25th percentile; p75, 75th percentile; E%, energy percentage; g, grams.

*Paired t-test or Wilcoxon matched-pair singed rank test for difference between baseline and end of intervention values.

Missing information on all nutrient variables: One participant.
