# Supplementary material for: Changes in adiposity, physical activity, cardiometabolic risk factors, diet, physical capacity and well-being in inactive women and men aged 57-74 years with obesity and cardiovascular risk – A 6-month complex lifestyle intervention with 6-month follow-up
Source: PLoS One. 2021 Aug 25;16(8):e0256631. doi: 10.1371/journal.pone.0256631 (PMC8386855; doi:10.1371/journal.pone.0256631)
Supplement: S3 Table — The RESTART pilot study 2017–18. (DOCX) [file pone.0256631.s004.docx]

**S3 Table.** **Change in daily food intake from baseline to end-of-intervention. The RESTART pilot study 2017-18.**

|  | Baseline | End of intervention | 95% CI/  p25, p75 | P-value* |
| --- | --- | --- | --- | --- |
| Vegetables, g/day | 196 (97) | 214 (158) | -47.27, 83.27 | 0.5636 |
| Fruit and berries, g/day | 492 (583) | 379 (380) | -143.1, 61.1 | 0.6496 |
| Fish and shellfish, g/day | 104 (71) | 111 (66) | -17.53, 30.46 | 0.5724 |
| Cakes, g/day | 12 (14) | 11 (12) | -5.09, 3.18 | 0.6287 |
| Sweets and sugar, g/day | 19 (19) | 20 (22) | -8.20, 9.40 | 0.8855 |
| Alcohol, g/day** | 11 (12) | 12 (11) | -5.30, 2.6 | 0.6892 |

Values are means (standard deviations) and confidence intervals or 25th and 75th percentiles for difference between measurements.

CI, confidence interval; p25, 25th percentile; p75, 75th percentile; E%, energy percentage; g, grams.

*Paired t-test or Wilcoxon matched-pair singed rank test for difference between baseline and end of intervention values.

**Alcohol nutrient intake (g/day).

Missing information on all dietary variables: One participant.
