# Supplementary material for: Changes in adiposity, physical activity, cardiometabolic risk factors, diet, physical capacity and well-being in inactive women and men aged 57-74 years with obesity and cardiovascular risk – A 6-month complex lifestyle intervention with 6-month follow-up
Source: PLoS One. 2021 Aug 25;16(8):e0256631. doi: 10.1371/journal.pone.0256631 (PMC8386855; doi:10.1371/journal.pone.0256631)
Supplement: S4 Table — The RESTART pilot study 2017–18. (DOCX) [file pone.0256631.s005.docx]

**S4 Table. Change in physical capacity from baseline to end-of-intervention. The RESTART feasibility study 2017-18.**

|  | Baseline | End of intervention | 95% CI/  p25, p75 | P-value* |
| --- | --- | --- | --- | --- |
| Resting heart rate, bpm | 74 (11) | 73 (14) | -5.85, 3.58 | 0.6144 |
| VO_2peak_, ml/kg/min | 25.1 (5.1) | 27.2 (6.6) | 0.14, 4.07 | 0.0381 |
| VO_2peak_, L/min | 2.64 (0.52) | 2.80 (0.69) | -0.03, 0.35 | 0.0923 |
| 1-RM chest press, kg | 42.1 (21.7) | 42.7 (19.6) | 0, 2.50 | 0.1093 |
| 1-RM lat pulldown, kg | 62.7 (14.8) | 68.0 (17.1) | 3.03, 7.68 | 0.0003 |
| 1-RM leg press, kg | 60.4 (33.5) | 137.5 (51.6) | 53.70, 100.62 | <0.0001 |

Values are means (standard deviations) and confidence intervals or 25^th^ and 75^th^ percentiles for difference between measurements.

CI, confidence interval, p25, 25th percentile; p75, 75th percentile; bpm, beats per minute, 1-RM, one repetition maximum, lat, lateral.

*Paired t-test or Wilcoxon matched-pair singed rank test for difference between baseline and end of intervention values. Following Benjamini–Hochberg adjustment, the change in VO_2peak_ was no longer statistically significant.

Missing information on VO_2peak_: Three participants.

Missing information on chest press: One participant.

Missing information on lat pulldown: Two participants.
