# Supplementary material for: Changes in adiposity, physical activity, cardiometabolic risk factors, diet, physical capacity and well-being in inactive women and men aged 57-74 years with obesity and cardiovascular risk – A 6-month complex lifestyle intervention with 6-month follow-up
Source: PLoS One. 2021 Aug 25;16(8):e0256631. doi: 10.1371/journal.pone.0256631 (PMC8386855; doi:10.1371/journal.pone.0256631)
Supplement: S5 Table — The RESTART pilot study 2017–18. (DOCX) [file pone.0256631.s006.docx]

**S5 Table.** **Change in psychological well-being from baseline to end-of-intervention. The RESTART pilot study 2017-18.**

|  | Cronbach’s alpha | Baseline | End of intervention | 95% CI | P-value* |
| --- | --- | --- | --- | --- | --- |
| Self-efficacy | 0.826; 0.910 | 3.11 (0.34) | 3.24 (0.46) | -0.27, 0.01 | 0.0727 |
| Self-esteem | 0.868; 0.726 | 3.32 (0.47) | 3.40 (0.42) | -0.21, 0.05 | 0.1998 |
| Satisfaction with life | 0.906; 0.977 | 5.26 (1.33) | 5.36 (1.54) | -0.51, 0.32 | 0.6295 |
| Anxiety/depression | 0.844; 0.855 | 1.28 (0.34) | 1.22 (0.34) | -0.06, 0.18 | 0.3193 |
| Global health | NA | 72.0 (13.62) | 77.83 (15.07) | -14.56, 2.89 | 0.1693 |

Values are means (standard deviations) and confidence intervals for difference between measurements.

CI, confidence interval.

Cronbach’s alpha, values from baseline; end-of-intervention.

Self-efficacy, the General Perceived Self-Efficacy Scale (10 questions, 4-graded scale); Self-esteem, Rosenberg’s Self-Esteem Scale (10 questions, 4-graded scale); Satisfaction with life, Satisfaction With Life Scale (3 questions, 7-graded scale); Anxiety/depression, Hopkins Symptom Checklist-10 (10 questions, 4-graded scale) reversed scale; Global health, modified EQ-VAS (0-100).

*Paired t-test for difference between baseline and end of intervention values.

Missing information on self-efficacy, self-esteem, satisfaction with life, anxiety/depression: Two participants.

Missing information on global health: Four participants.
