# Supplementary material for: Changes in adiposity, physical activity, cardiometabolic risk factors, diet, physical capacity and well-being in inactive women and men aged 57-74 years with obesity and cardiovascular risk – A 6-month complex lifestyle intervention with 6-month follow-up
Source: PLoS One. 2021 Aug 25;16(8):e0256631. doi: 10.1371/journal.pone.0256631 (PMC8386855; doi:10.1371/journal.pone.0256631)
Supplement: S1 Appendix — (PDF) [file pone.0256631.s007.pdf]

Tools for Lasting Lifestyle Changes

Protocol

(short version)

Pilot study

Autumn 2017 – Spring 2018

Original version May 9th 2017, short version August 15th 2020

Content

Summary 3

Background 4

Purpose and target group 4

Population 4

Aim 5

Research questions 5

Sample and recruitment 5

Intervention 6

Exercise 6

Diet 6

Psychology 7

Analysis 7

Feasibility 7

Participants' experiences 7

Effect of intervention 7

Testing of activity trackers 8

Activities 10

Ethics and user participation 10

References 11

Summary

There is limited research-based knowledge about individual-oriented interventions that can contribute to lasting lifestyle changes in inactive middle-aged and elderly people with obesity and an increased risk of cardiovascular disease. Intervention studies with long-term follow-up are important for measuring the effect and maintenance of the effect of lifestyle interventions. Our goal is to develop a complex lifestyle intervention with exercise, nutritional counselling, and psychology counselling that will provide a lasting lifestyle change in inactive middle-aged and older men and women with obesity and increased risk of cardiovascular disease. In development of intervention studies, it is important to carry out pilot studies to investigate feasibility and intervention effects. This study is a pilot and feasibility study designed to investigate the study feasibility, participants' experiences, the effect of the intervention, as well as testing the use of activity trackers for data collection (Table 1).

Table 1. Summary of the elements of the pilot study.

**Purpose of the study**	Pilot study: Evaluation of feasibility, participants' experiences, effect of intervention, and testing of activity trackers
**Study population**	Tromsø 7 participants that are middle aged/elderly, physically inactive, have obesity and increased risk of cardiovascular disease
**Study design**	One group (the main study is planned as a randomized controlled trial)
**Evaluation of feasibility**	Assessment of recruitment, logistics, intervention elements, dropouts and compliance
**Exploration of participant experiences**	Analysis of participant experiences (qualitative interviews, focus group interviews)
**Analysis of the intervention effect**	Analysis of intervention effect: Adiposity (body mass index, waist- and hip circumference, body composition), physical activity (accelerometer), cardio-metabolic risk factors (blood pressure, heart rate, blood lipids, HbA1c), diet (food and nutrients), physical capacity (strength, endurance) , health-related quality of life (questionnaire)
**Testing of activity trackers**	Analysis of validity of instruments (validation studies), analysis of user experiences (qualitative interviews)
**Intervention**	Exercise (1-hour group exercises 2 times per week) based on endurance (bike spinning) and strength/balance/flexibility (body weight/dumbbell exercises, strength training) training led by physiotherapists<br>Diet (1 individual and 3 2-hour group counselling/teaching (general and practical food knowledge, shopping and cooking) sessions) led by nutritionist<br>Psychology (3 group counselling (self-regulation, implementation-intention strategies) sessions) led by psychologist
**Treatment group**	26 weeks (from baseline screening to end of intervention)
**Control group**	No
**Duration of treatment**	22 weeks (intervention period)
**Duration of observation time**	26 weeks (from baseline to end of intervention)
**Duration of total follow-up**	52 weeks (baseline to end of follow-up)
**Number of study participants included for evaluation**	12-20 (minimum of 12 participants)
**Number of study participants recruited**	12-20 (minimum of 12 participants)

Background

There is limited research-based knowledge about individual-oriented interventions that can contribute to **lasting lifestyle changes** in inactive people with obesity and an increased risk of cardiovascular disease. Increase in obesity and sedentary behaviour are public health in both Norway and globally (1-3). Obesity increase the risk of diabetes, cardiovascular disease and death (4) and reduce mobility, is associated with poorer self-control, depression and poorer appetite control (5). A sedentary lifestyle increases the risk of obesity, cardiovascular disease and premature death (6).

Overweight and obesity are increasing in Norway (7, 8) and in 2007-2008, one in five participants in the Tromsø Study was obese. **Sitting** at work has increased significantly since the 1970s (9). The recommendation of 150 minutes per week of physical activity in moderate intensity can provide significant health benefits for both the individual and society, but in a Norwegian report from 2014-2015, only 32% met the national physical activity recommendations (3). **Exercise** increases endurance, strength and mobility, and exercises programs combining these elements are particularly beneficial (10, 11). While sedentary behaviour has been shown to increase the risk of cardiovascular disease (12), physical activity and exercise contribute to reduced risk (13). **Diet** is the leading risk factor in Norway associated with the most lost life years lost and days of illness and injury (14). Population intake of vegetables, fruit, berries, whole grains and fish is lower than Norwegian Directorate of Health's recommendations, while intakes of fibre and saturated fat is too high (15, 16). Implementation of lifestyle changes can be challenging. Self-regulation strategies like Implementation Intentions have proven to be effective to **health behaviour** changes, including dietary changes (17). Implementation Intentions strategies are based on specific situations that the individual expects to be challenging, to where the individual formulate explicit strategies for how the situation can be handled, based on “If-then” planning (18).

It is important that interventions to reduce risk factors must aim at long-term maintenance of lifestyle. While many overweight people achieve weight loss after a time-limited lifestyle change, **maintenance of lifestyle change** is challenging (19). There is evidence that **complex interventions** consisting of both exercise and diet intervention elements are more effective than interventions consisting of only one element (20), and it may also seem that complex interventions including both exercise, diet and behavioural elements (21) provide increased long-term effect. Therefore, there is a need for **development and testing of complex lifestyle interventions aimed at long-term maintenance of favourable lifestyle changes**.

Purpose and target group

Population

The Tromsø Study (22) is an ongoing population study, to study the causes, risk factors, prevention and treatment of diseases in the general population. The Tromsø Study started in 1974, and is Norway's longest ongoing and most comprehensive collection of health data. The data collection includes questionnaires, biological samples and clinical examinations. Findings provide the basis for research questions and hypotheses to be tested in intervention studies, where participants are recruited on the basis data in the Tromsø Study. In the seventh Tromsø Study (Tromsø 7 2015-2016), all women and men 40 years and older living in the Tromsø municipality were invited, and 21,083 (65%) participated (22). The comprehensive data material provides a good basis for the development of targeted preventive measures and interventions.

Aim

The aim of the study is to develop a lifestyle intervention to be tested in a randomized controlled trial (RCT). We will design and test the long-term effects of a complex lifestyle intervention (exercise, dietary and psychology counselling) aimed at middle-aged and elderly people with obesity and increased risk of cardiovascular disease. When developing intervention studies, it is important to carry out pilot studies to investigate feasibility. Thus, this project is a pilot study with the aim of examining study feasibility, participants' experiences, the effect of the intervention, and testing of activity trackers for use in the data collection. Study participants are recruited from Tromsø 7 on the basis of self-reported inactivity, measured obesity and elevated cardiovascular disease risk. The pilot study will be carried out as an open study. Experiences from the pilot study will be important for the development of the main study (the RCT).

Research questions

1. Evaluate study feasibility
   a. Analysis of recruitment, logistics, treatment arrangements, dropouts, compliance
2. Explore participants' experiences
   a. Analysis of participants' experiences using qualitative interviews and focus group interviews
3. Measuring the effect of intervention (therapeutic response)
   a. Adiposity (body mass index, waist and hip circumference, body composition), physical activity (accelerometer), cardiovascular risk factors (blood pressure, heartrate, blood lipids, HbA1c), diet (food and nutrient intake calculated from food frequency questionnaires), endurance and strength (maximal oxygen uptake , strength training), health-related quality of life (questionnaires)
4. Testing of activity trackers
   a. Analysis of validity of instruments (validation studies)
   b. Analysis of participants' experiences including user friendliness (qualitative interviews)

Sample and recruitment

Inclusion and exclusion criteria

Inclusion criteria are age (55-75 years), body mass index ≥30 kg/m^2, elevated and elevated NORRISK2 score. Exclusion criteria are previous myocardial infarction, severe illness with short life expectancy, disease, condition or functional level (including ECG findings, blood pressure measurements or blood tests, as well as physical function tests) or practicalities (including inability to participate in weekly afternoon exercise seessions) that limits participation. Potential participants may have little or no experience with training and may find it group exercise challenging. We have access to special premises to be used for screening and for the exercise intervention.

Invitation and screening

A random sample of 70 participants drawn from the Tromsø 7 sample who fit the inclusion criteria are invited to participate. A letter of invitation including study information will be sent by mail. People responding will be interviewed with respect to the inclusion and exclusion criteria. The goal is to include 12-20 participants (minimum 12 participants, based on the size of a standard gym exercise group). Planned media publicity (local newspaper, local radio, Science Week contributions) may aid recruitment.

Intervention

The pilot study will be conducted as an open study including 12-20 participants recruited from the seventh Tromsø Study to a 22-week intervention period without control group. Participants will undergo an intervention consisting of exercise, and dietary and psychology counselling. Participants will wear an activity tracker that records all physical activity continuously, from baseline to end of follow-up. The participants will be followed up 6 months after end of intervention. Measurements to be conducted at baseline, midway, end of intervention, and end of follow-up are described below (as well as in Table 2).

Exercise

Measurement method

Physical activity will be measured with an accelerometer (ActiGraph wGT3X-BT) (at baseline and end of intervention) and with an activity tracker (Polar M430) (continuously from baseline to end of follow-up). The activity tracker will be used as an endpoint assessment and no feedback will be given to the study participant.

Intervention

The exercise will be carried out in groups (maximum of 20 participants). The exercise will be organized as a 1-hour workout two days (Tuesday, Thursday) per week, parted into 3 different sections. First a 6- week section with strength training on day 1 and endurance exercise on day 2. In the next 6-week section, both days will contain elements from both strength and endurance exercise, while in the last 12-week section, the exercise will be conducted as section 2 but with higher exercise intensity. The exercise intensity for parts of section 1 and section 2 will be 80% or more of the maximum heart rate and for section 3 it is planned that parts of the sessions will take place at a minimum above 85% of the maximum heart rate. Exercise intensity is controlled with the participants' heart rate monitors (Polar RS400) and based on the participants' maximum heart rate from the VO_{2max}-test. The exercise sessions will mainly take place indoors in the gym with various exercises such as bike spinning, running , core-pulse etc, but there will also be some outdoor exercises. Strength training is included in the form of varied exercises with up to 8 repetitions in groups in the gym. Leg press is included as a special strength exercise before/after the sessions as a 3x5 series. Two dedicated personal trainers/fitness instructors with physiotherapist education will lead the exercise sessions.

Diet

Measurement method

Food habits and nutrient intake will be measured by a 3-day 24-hour food diary (at baseline) and a food frequency questionnaire (at baseline and end of intervention). Food intake and nutrients will be calculated based on the food frequency questionnaire.

Intervention

Dietary counselling will be conducted as one individual counselling and two group lessons of 2 hours duration each. Prior to the individual dietary counselling, the participant completes a 3-day food diary. The group lessons (maximum 8 participants per group) consists of the themes food knowledge, shopping and cooking, based on the national dietary guidelines. A dedicated nutritionist will conduct all dietary counselling and teaching.

Psychology

Measurement method

Health-related quality of life (self-efficacy, self-esteem, satisfaction with life, symptoms of anxiety and depression, and self-reported health) is measured with a questionnaire at baseline, midway and end of intervention.

Intervention

The psychology course are conducted in groups (maximum 8 participants per group) of 1 hour, 3 times. The course includes an introduction to lifestyle change, motivation, eating behaviour, thoughts and feelings, and everyday planning. The psychologist will present examples of typical challenges, the important characteristics of a critical situation and a particular behaviour is demonstrated with examples. Participants are instructed to form 2-3 "if-then" strategies: First, they should think about the goal that is most critical to their personal situation. Then they should identify 2-3 actions they could do that would bring them closer to achieving this goal. These should be 2-3 situations in everyday life that are good situations to perform these actions. The psychologist will assess whether the participants' situations and actions are suitable for the "if-then" strategy. Finally, participants will write down these critical situations and the intended action in an "if-then" plan format and commit to the plans. A dedicated psychologist will conduct the teaching.

Analysis

Analysis of feasibility, participant's experiences, intervention effect and testing of activity trackers will be conducted after the intervention/end of follow-up with the use of different methods described below (as well as in Table 2).

Feasibility

Feasibility is evaluated with various goals and measurement methods, including recruitment (including assessment of response rate), logistics from screening to end of intervention, dropout and compliance (including dropout from the study, attendance at exercise sessions, dietary guidance sessions and psychology session, and activity tracker wear-time), and registration of adverse events/injuries/illness.

Participants' experiences

Participants' experiences are explored using qualitative methods. The purpose is to study the experiences with the various intervention elements, and to uncover potentials for improvement for the main study. Analysis of participants' experiences is performed with individual qualitative interviews and focus group interviews. An interview guide will be developed.

Effect of intervention

The effect of the intervention will be measured by change in measurements of adiposity (body mass index, waist and hip circumference, body composition), physical activity level (accelerometer), cardiovascular risk factors (blood pressure, heart rate, blood lipids, HbA1c), diet (nutrient calculations from the food frequency questionnaire), endurance and strength (VO_{2max}, strength exercises), and health-related quality of life (questionnaires).

Testing of activity trackers

Activity meters will be tested and examined in relation to the validity of the instrument (validation study of Polar M430 compared with ActiGraph wGT3X-BT) and an analysis of participant experiences/user friendliness (qualitative interviews).

Table 2. Tests performed at baseline/midway/end of intervention/end of follow-up.

**Goal**	**Test description**
Adiposity	
Body mass index	Height and weight measurement at baseline, midway and end of intervention.
Waist and hip circumference	Waist- and hip circumference measurement at baseline, midway and end of intervention.
Body composition	Fat- and lean mass measured by DEXA, at baseline, midway and end of intervention.
Ideal weight	Questionnaire at baseline, midway and end of intervention.
Physical activity	
Physical activity	Accelerometer measurements (number of steps, minutes in sedentary, light moderate and vigorous activity, energy expenditure, etc) will be measured with ActiGraph wGT3X-BT at baseline and end of intervention and with Polar M430 continuously from baseline to end of follow-up, and self-reported activity and sitting, at work and leisure time with questionnaire at baseline, midway and end of intervention.
Cardiometabolic risk factors	
Blood pressure and heart rate	Systolic and diastolic blood pressure as well as resting heart rate will be measured at baseline, midway and end of intervention.
Biomarkers in blood and urine	Blood samples will be analysed for total cholesterol, LDL cholesterol, HDL cholesterol, triglycerides, creatinine, CK, ALAT, ASAT, GT, 25-OH-vitaminD, 25-OH-vitaminD2, 25-OH-vitaminD3, HbA1c, glucose and haemoglobin, at baseline, midway and end of intervention, and FT4 and THS at baseline. Urine samples will be analyzed for albumin-creatinine ratio at baseline, midway, and end of intervention.
Smoking	Smoking status will be measured by questionnaire at baseline, midway and end of intervention.
Heart parameters	ECG at baseline, midway and end of intervention.
Diet	
Food habits	3-day food diary at baseline, and a food frequency questionnaire at baseline and intervention end.
Food intake/energy intake/nutrient intake	(From the food frequency questionnaire).
Physical capacity	
Lung function	Spirometry at baseline and end of intervention.
Range of motion	Movement is measured for neck, shoulder, hip and ankle, at baseline and end of intervention.
Strength	Muscle strength will be tested in 4 basic exercises (leg press, chest press, shoulder press and pull-down) at baseline, midway and end of intervention. All exercises will be tested with 1 repetition (1 RM). In addition, power in leg press (Rate of Force Development) will be tested.
Balance	Balance (standing balance, eyes closed) is measured with the same protocol as in Tromsø 7 (the time the participants manage to stand on one leg with eyes closed). Tested at baseline, midway and end of intervention.
Endurance	Maximum oxygen uptake (VO_{2max}) and maximum heart rate are measured on a treadmill with a mask and heart rate monitor, at baseline and end of intervention.
Health-related quality of life	

Self-efficacy	Questionnaire at baseline, midway and end of intervention.
Self-esteem	Questionnaire at baseline, midway and end of intervention.
Satisfaction with life	Questionnaire at baseline, midway and end of intervention.
Symptoms of anxiety/depression	Questionnaire at baseline, midway and end of intervention.
Self-reported health	Questionnaire at baseline, midway and end of intervention.
Participant experience	
Experiences related to lifestyle change (intervention)	Attitudes, motivation, barriers to lifestyle changes with a qualitative interview midway and end of follow-up.
Experiences related to the intervention	Experiences of participation and experience using the activity tracker, with focus group interviews at end of intervention.
Other measures	
Demographic data, medical history, etc.	Various demographic data (education, work) and medical history (disease, diseases in the family, conditions, level of function, medication use, pain, sleep, social relationships) with questionnaire at baseline, midway and end of intervention.

Activities

The overall timetable for the planned study is shown in Table 3, details for activities in Table 4 . Invitations will be sent out in August 2017, screening and baseline measurements carried out in September 2018, the intervention period will range from October 2017 to March 2018, and end of follow-up will be September 2018.

Table 3. Time plan of the pilot study.

Aktivitet	aug.17	sep.17	okt.17	nov.17	des.17	jan.18	feb.18	mar.18
Rekruttering								
Intervju deltakere								
Basismålinger								
Oppstart intervensjonen								
Intervensjons periode								
Registrering								

Ethics and user participation

Participants will signed consent form after having received information about the study. We will apply REK for ethical approval. The project's steering group includes two user representatives, from a patient association and the municipality, who contribute with experiences from the patient perspective, and from experiences with the conduction of a complex intervention performed in the municipality, respectively.

References

1. Hallal PC, Andersen LB, Bull FC, Guthold R, Haskell W, Ekelund U. Global physical activity levels: surveillance progress, pitfalls, and prospects. Lancet. 2012;380:247-57.
2. World Health Organization. Global status report on noncommunicable diseases 2010. Geneva: World Health Organization; 2011.
3. Norweigan Institute of Public Health. Folkehelserapporten 2014. Oslo, Norway: National Institute of Public Health
2014 06.30.2014.
4. Flegal KM, Kit BK, Orpana H, Graubard BI. Association of all-cause mortality with overweight and obesity using standard body mass index categories: a systematic review and meta-analysis. JAMA. 2013;309:71-82.
5. Danielsen KK, Sundgot-Borgen J, Maehlum S, Svendsen M. Beyond weight reduction: improvements in quality of life after an intensive lifestyle intervention in subjects with severe obesity. Ann Medicine. 2014;46:273-82.
6. Kokkinos P. Physical activity, health benefits, and mortality risk. ISRN Cardiology. 2012;2012:718789.
7. Jacobsen BK, Aars NA. Changes in body mass index and the prevalence of obesity during 1994-2008: repeated cross-sectional surveys and longitudinal analyses. The Tromso Study. BMJ Open. 2015;5:e007859.
8. Groholt EK, Stigum H, Nordhagen R. Overweight and obesity among adolescents in Norway: cultural and socio-economic differences. J Public Health. 2008;30:258-65.
9. Morseth B, Jacobsen BK, Emaus N, Wilsgaard T, Jorgensen L. Secular trends and correlates of physical activity: The Tromso Study 1979-2008. BMC Public Health. 2016;16:1215.
10. Karlsen T, Helgerud J, Stoylen A, Lauritsen N, Hoff J. Maximal strength training restores walking mechanical efficiency in heart patients. Int J Sports Medicine. 2009;30:337-42.
11. Heggelund J, Fimland MS, Helgerud J, Hoff J. Maximal strength training improves work economy, rate of force development and maximal strength more than conventional strength training. Eur J Applied Physiol. 2013;113:1565-73.
12. Yusuf S, Hawken S, Ounpuu S, Dans T, Avezum A, Lanas F, et al. Effect of potentially modifiable risk factors associated with myocardial infarction in 52 countries (the INTERHEART study): case-control study. Lancet. 2004;364:937-52.
13. Nocon M, Hiemann T, Muller-Riemenschneider F, Thalau F, Roll S, Willich SN. Association of physical activity with all-cause and cardiovascular mortality: a systematic review and meta-analysis. Eur J Cardiovasc Prevent Rehab. 2008;15:239-46.
14. Institute for Health Metrics and Evaluation. Global Burden of Diseases, Injuries, and Risk Factors Study 2010. Available from:
http://www.healthdata.org/sites/default/files/files/country_profiles/GBD/ihme_gbd_country_report_norway.pdf
15. Totland TH, Melnæs BK, Hallen NL, Kigen KMH, L. BNA, B. MJ. Norkost 3. En landsomfattende kostholdsundersøkelse blant menn og kvinner i Norge i alderen 18-70 år, 2010-11 Oslo. 2012. Report No.: IS-2000.
16. Utviklingen i norsk kosthold 2014. Oslo: Helsedirektoratet; 2015. Report No.: IS-2255.
17. Adriaanse MA, Vinkers CD, De Ridder DT, Hox JJ, De Wit JB. Do implementation intentions help to eat a healthy diet? A systematic review and meta-analysis of the empirical evidence. Appetite. 2011;56:183-93.
18. M. GP. Implementation intentions: Strong effects of simple plans. Am Psychologist. 1999;54:10.
19. Wing RR, Phelan S. Long-term weight loss maintenance. Am J Clin Nutr. 2005;82:222s-5s.
20. Wu T, Gao X, Chen M, van Dam RM. Long-term effectiveness of diet-plus-exercise interventions vs. diet-only interventions for weight loss: a meta-analysis. Obesity Reviews. 2009;10:313-23.
21. Dombrowski SU, Knittle K, Avenell A, Araujo-Soares V, Sniehotta FF. Long term maintenance of weight loss with non-surgical interventions in obese adults: systematic review and meta-analyses of randomised controlled trials. BMJ. 2014;348:g2646.
22. Jacobsen BK, Eggen AE, Mathiesen EB, Wilsgaard T, Njølstad I. Cohort profile: the Tromsø Study. Int J Epidemiol. 2012;41:961-967.

Table 4. Activity plan.

AKTIVITETER	Q4			Q1			Q2			Q3			Q4			Q1		
	okt.16	nov.16	des.16	jan.17	feb.17	mar.17	apr.17	mai.17	jun.17	jul.17	aug.17	sep.17	okt.17	nov.17	des.17	jan.18	feb.18	mar.18
**ADMINISTRASJON OG LEDELSE -**																		
Rekruttere Prosjektleder																		
Oppstart Prosjektleder	■																	
Prosjektorganisering	■	■	■	■														
Budsjett revisjon	■	■	■	■			■					■			■			
Prosjektbeskrivelse utformes		■	■	■	■	■	■											
Utarbeidelse av oppdatert protokoll		■	■	■	■	■	■											
Utarbeidelse av oppdatert aktivitetsplan		■	■	■	■													
Etablering av basisorganisasjon	■	■																
Kartlegging ressurspersoner og faglig netverk	■	■	■	■	■	■	■											
Møter prosjekt og arbeidsgrupper - behovstilpasses	■	■	■	■	■	■	■	■	■	■	■	■	■	■	■	■	■	■
Etablering av styringsgruppen		■	■	■														
Møter styringsgruppen					■				■				■					■
Avklare inklusjonskriterier				■	■	■												
Søke forskningsposten samarbeid pilot/prosjekt				■	■													
Ev. søke Alfheim samarbeide pilot							■											
Etablering av faglig nettverk		■	■	■	■	■	■											
Forberede workshop				■	■	■	■											
Arrangere workshop for pilot og hovedprosjekt							■										■	
Søknad REK								■										
Planlegge pilot Stamina					■	■	■	■	■									
Etablering referansegruppe for hovedprosjekt							■	■	■									
Søknader finansiering hovedprosjekt							■	■	■	■	■	■	■	■	■	■	■	■
Lage invitasjonsbrev deltakere									■									
Forsikring deltakere						■						■						
Kick-off Pilotorganisasjonen (fellesmiddag?)						■						■						
Oppstart Pilot (Deltakere)													■					
Underveisevaluering Pilot															■	■		
Avslutning Pilot (Deltakere)																		■
Evaluering Pilot																		■

AKTIVITETER	Q4			Q1			Q2			Q3			Q4			Q1		
	okt.16	nov.16	des.16	jan.17	feb.17	mar.17	apr.17	mai.17	jun.17	jul.17	aug.17	sep.17	okt.17	nov.17	des.17	jan.18	feb.18	mar.18
**VITENSKAPELIG PROFIL**																		
REK protokoll og invitasjon ferdigstilles						■	■											
Utsjekking ift Datatilsynet							■											
Analyser og tilbakemelding til deltakere																		■
**INFRASTRUKTUR & LOGISTIKK**																		
Avklare lokaler til bruk i pilot				■	■	■												
Avklar blodprøvetaking underveis i pilot					■						■	■	■	■				
Kontraktsinngåelse - Stamina/Alfheim/Forskn.post						■	■	■	■	■								
Klargjøre Alfheim/Forskningspost/ Stamina											■	■	■	■				
Logistikk stasjoner									■	■	■	■	■	■				
Infrastruktur (møbler, logistikk etc)									■	■	■	■	■	■	■	■	■	■
**EUTRO / IT**																		
Utvikle prosjektets IT løsning							■	■	■							■	■	■
Oppmøtedatabase										■	■	■						
Risikovurdering av informasjonssikkerhet og personvern ?								■										
Klargjøre lagring blodprøver/Analysesvar Bio							■	■										
**LAB / BIOBANK / HMS**																		
Internkontrollsystem og avvikshåndtering							■	■	■	■	■	■	■	■	■	■	■	■
**ØKONOMI / INNKJØP**																		
Budsjett	■	■	■	■				■				■			■			■
Regnskapsoppbygging Agresso	■	■																
Søknadsskriving - finansiering		■	■	■	■	■	■	■	■	■	■	■	■	■	■	■		
Årsregnskap og rapporteringer			■	■											■	■		
Økonomistyring	■	■	■	■	■	■	■	■	■	■	■	■	■	■	■	■	■	■
**FORMIDLING**																		
Formidlingsstrategi og plan						■	■	■	■									
Fotoprosjekt												■	■	■	■	■		
Forskningstorget																		
Nyhetsbrev									■	■	■	■	■	■	■	■	■	■
Ekstern og intern kommunikasjon	■	■	■	■	■	■	■	■	■	■	■	■	■	■	■	■	■	■
